# Investigation of the Effects of Curcumin on Serum Cytokines in Obese Individuals: A Randomized Controlled Trial

**DOI:** 10.1155/2014/898361

**Published:** 2014-02-11

**Authors:** Shiva Ganjali, Amirhossein Sahebkar, Elahe Mahdipour, Khadijeh Jamialahmadi, Sepideh Torabi, Saeed Akhlaghi, Gordon Ferns, Seyed Mohammad Reza Parizadeh, Majid Ghayour-Mobarhan

**Affiliations:** ^1^Cardiovascular Research Center, Mashhad University of Medical Sciences, Mashhad, Iran; ^2^Department of Biotechnology, Science & Research Branch, Islamic Azad University, Tehran, Iran; ^3^Biotechnology Research Center, Mashhad University of Medical Sciences, Mashhad, Iran; ^4^Neurogenic Inflammation Research Centre, Department of Medical Biotechnology, Mashhad University of Medical Sciences, Mashhad, Iran; ^5^Department of Medical Biotechnology, School of Medicine, Mashhad University of Medical Sciences, Mashhad, Iran; ^6^Department of Plant Breeding, Science & Research Branch, Islamic Azad University, Tehran, Iran; ^7^Deputy of Research, Faculty of Medicine, Mashhad University of Medical Sciences, Mashhad, Iran; ^8^Division of Medical Education, Mayfield House, University of Brighton, Room 342, Brighton BN1 9PH, UK; ^9^Biochemistry of Nutrition Research Center, Mashhad University of Medical Sciences, Mashhad, Iran

## Abstract

*Background*. Obesity is a disorder often accompanied by a heightened state of systemic inflammation and immunoactivation. The present randomized crossover trial aimed to investigate the efficacy of curcumin, a bioactive polyphenol with established anti-inflammatory and immunomodulatory effects, on the serum levels of a panel of cytokines and mediators in obese individuals. *Methods*. Thirty obese individuals were randomized to receive curcumin at a daily dose of 1 g or a matched placebo for 4 weeks. Following a 2-week wash-out period, each group was assigned to the alternate treatment regimen for another 4 weeks. Serum samples were collected at the start and end of each study period. Serum levels of IL-1**α**, IL-1**β**, IL-2, IL-4, IL-6, IL-8, IL-10, VEGF, IFN**γ**, EGF, MCP-1, and TNF**α** were measured using a multiplex Biochip Array Technology based method. *Results*. Mean serum IL-1**β** (*P* = 0.042), IL-4 (*P* = 0.008), and VEGF (*P* = 0.01) were found to be significantly reduced by curcumin therapy. In contrast, no significant difference was observed in the concentrations of IL-2, IL-6, IL-8, IL-10, IFN**γ**, EGF, and MCP-1. *Conclusions*. The findings of the present trial suggested that curcumin may exert immunomodulatory effects via altering the circulating concentrations of IL-1**β**, IL-4, and VEGF.

## 1. Introduction

Obesity is a global health problem and is increasing in prevalence (~60%) over the past 20 years. According to the World Health Organization (WHO) statistics, there were ~1.6 billion overweight adults globally, of whom about 400 millions were obese in 2005 [1]. The list of comorbidities associated with obesity is extensive, among which is cardiovascular disease [2]. A plethora of scientific evidence has confirmed the predisposing effects of obesity on the development and progression of atherosclerosis as well as the risk of other coronary risk factors including hypertension, type 2 diabetes, and dyslipidemia. Obesity is associated with a strong inflammatory response and is often accompanied by increased levels of proinflammatory cytokines and impaired antioxidant status [3]. The most important source of proinflammatory cytokines in obesity is macrophages that infiltrate adipose tissue as a response to the adipocyte growth, decreased blood supply, hypoxia, and tissue necrosis. These events collectively predispose to a systemic inflammation which is itself a triggering factor for the pathogenesis of obesity related morbidities [4].

Cytokines are small molecules with protein or glycoprotein structure (8–80 kDa). They are products of activated immune cells that act as molecular signals between immune competent cells [5]. Among cytokines, interleukin 1 (IL-1), tumor necrosis factor-*α* (TNF*α*), and interleukin-6 (IL-6) are major inducers of acute phase response [6].

During recent decades, natural products have attracted considerable attention as antiobesity agents. Curcumin is the bioactive yellowish pigment of turmeric and is amongst the most promising natural products. Curcumin has numerous health benefits [7–18] and has been shown to interact with a wide range of molecular targets including transcription factors, cytokines, protein kinases, growth factors, cell adhesion molecules, redox state enzymes, and receptors [19, 20]. The anti-inflammatory effects of curcumin are well established and are due in part to its effects on the activity of cyclooxygenase-2 (COX-2), lipoxygenase, and inducible nitric oxide synthase (iNOS) enzymes and inhibition of inflammatory cytokine (such as TNF-*α*, monocyte chemoattractant protein 1 (MCP-1), and interleukins 1, 2, 6, 8 and 12) production [21]. The present study was undertaken to assess the influence of supplementation with curcuminoids on serum levels of inflammatory cytokines in obese individuals.

## 2. Materials and Methods

### 2.1. Subjects

The present study was performed on serum samples from our previous trial that investigated the hypolipidemic effects of curcumin in obese individuals. Details of the aforementioned study have been described elsewhere [8]. Briefly, thirty-seven patients were recruited and provided written informed consent. Seven participants dropped out of the trial due to reported constipation (*n* = 3), bloating (*n* = 3), and increased frequency of urination (*n* = 1).

Inclusion criteria were obese subjects (with body mass index (BMI) ≥ 30) who had either < 2 risk factors (except diabetes mellitus) for coronary heart disease (CHD) plus 160 mg/dL < LDL-C < 190 mg/dL or ≥ 2 CHD risk factors (except diabetes mellitus) and 130 mg/dL < LDL-C < 160 mg/dL. Exclusion criteria were history of systemic diseases (such as systemic lupus erythematosus, kidney disease, diabetes mellitus, and established cardiovascular disease), consumption of drug supplements within the preceding 6 months, and history of taking any lipid-lowering drug. Thirty-seven subjects (aged 18–65 years) fulfilled the inclusion criteria and entered the trial. The study protocol was approved by the Ethics Committee of the Mashhad University of Medical Sciences and all participants provided a written informed consent.

### 2.2. Drugs

C3 Complex formula (obtained from Sami Labs Ltd, Bangalore, India) was used as the source of curcuminoids (comprising curcumin, demethoxycurcumin, and bisdemethoxycurcumin) for the present study. In order to enhance the bioavailability of curcminoids, which is usually the main concern for the lack of efficacy, a coadministration strategy with bioperine (Sami Labs Ltd, Bangalore, India) was applied. Curcuminoids were administered in the form of hard gelatin capsules containing 500 mg C3 Complex plus 5 mg bioperine. The placebo capsules matched the active capsules in size and shape and contained bioperine (5 mg) alone.

### 2.3. Study Design

This was a randomized, double blind, crossover trial in which each patient received curcuminoids (1 g/day) or placebo and then crossed over to the alternate regimen. Each treatment period was 30 days and there was a 2-week wash-out interval between the regimens.

### 2.4. Measurement of Cytokines

Cytokines measurements were performed by using the Biochip Array Technology on the Randox Evidence Investigator (Randox Laboratories, Belfast, Northern Ireland). The Evidence Investigator Biochip Array Technology is used to perform simultaneous quantitative detection of multiple analytes from a single patient sample. The applied cytokine array biochip employs a sandwich chemiluminescent immunoassay for a high throughput measurement of circulating cytokines. The light signal generated from each of the test regions on the biochip is detected using digital imaging technology and compared to that from a stored calibration curve. The concentration of cytokines present in the sample is calculated from the calibration curve. The Evidence Investigator Cytokine Array can simultaneously determine the concentrations of IL-1*α*, IL-1*β*, IL-2, IL-4, IL-6, IL-8, IL-10, vascular endothelial growth factor (VEGF), interferon *γ* (IFN*γ*), epidermal growth factor (EGF), MCP-1, and TNF*α*.

### 2.5. Statistical Analysis

All analyses were performed with the statistical analysis software (SAS; version 9.1). A mixed model analysis of variance for 2 × 2 crossover studies was fitted when assumption for normality was met. A two-sided *P* value of < 0.05 was considered as statistically significant.

## 3. Results

There were significant period effects from the first period to the second period of study for VEGF (*P* = 0.01) and no significant period effect for all of the remaining parameters including IL-1*α*, IL-1*β*, IL-2, IL-4, IL-6, IL-8, IL-10, IFN*γ*, MCP-1, EGF, and TNF*α* (*P* > 0.05). Dietary supplementation with curcuminoids was found to be associated with significantly reduced serum levels of IL-1*β* (*P* = 0.042), IL-4 (*P* = 0.008), and VEGF (*P* = 0.03). On the other hand, curcuminoids supplementation did not alter serum concentrations of IL-1*α*, IL-2, IL-6, IL-8, IL-10, IFN*γ*, EGF, MCP-1, and TNF*α* (*P* > 0.05). The mean cytokines concentrations of the two groups of participants in each period of study are shown in Table 1.

## 4. Discussion

The present trial demonstrated that, in obese patients, supplementation with curcumin (1 g/day) results in a significant decline in serum levels of IL-1*β*, VEGF, and IL-4, while having no impact on the concentrations of IL-1*α*, IL-2, IL-6, IL-8, IL-10, IFN*γ*, EGF, MCP-1, and TNF*α*.

Systemic inflammation is the common feature of obesity, cardiovascular disease, and diabetes mellitus, linking these disorders to each other. Proof-of-concept studies have shown that heightened state of inflammation associated with obesity stems from a delicate crosstalk between adipocytes and macrophages which involves infiltration of macrophages to the adipose tissue, NF-*κ*B activation, and increased expression and release of a panel of inflammatory cytokines [22–24].

Modulation of inflammatory response is among the biological effects of curcumin. Numerous *in vitro*, *in vivo*, and clinical studies have endorsed the anti-inflammatory properties of curcumin as reviewed elsewhere [25]. These anti-inflammatory effects are exerted via different mechanisms due to the interactions of curcumin with a wide range of biomolecules of different classes such as transcription factors, cellular receptors, growth factors, enzymes, cytokines and chemokines. Downregulation of NF-*κ*B has been proposed as the most celebrated mechanism for the medicinal properties of curcumin. NF-*κ*B plays a significant role in the regulation of transcription of several vital inflammatory mediators including inducible nitric oxide synthase (iNOS), cyclooxygenase-2 (COX-2), TNF*α*, IL-6, and IL-8. Inhibition of NF-*κ*B activation has been reported to account for the blunting effects of curcumin on inflammatory cytokine expression and release in both preadipocytes and differentiated 3T3-L1 adipocytes [24]. Anti-inflammatory properties of curcumin can also be exerted as a result of 5-lipoxygenase and p38 mitogen-activated protein kinase (MAPK) inhibition, as well as mitigation of Janus kinase-STAT inflammatory signaling pathway [26].

In the present trial, curcumin reduced serum levels of VEGF, IL-1*β*, and IL-4. VEGF overexpression and subsequent vasculogenesis and angiogenesis are implicated in the development of several pathological processes. Some important examples include chronic inflammatory disorders and different types of carcinomas [27]. On the other hand, mounting evidence has shown that blockade of VEGF response is of therapeutic utility in several inflammatory disorders and malignancies [28]. In previous investigations, curcumin and its structural analogues have been shown to downregulate expression, secretion, and biological activities of VEGF and its receptor [27, 29, 30]. The findings on the modulatory effects of curcuminoids on serum levels of IL-1*β* and IL-4 are consistent with those previously reported *in vitro *and *in vivo *[31–34]. These effects could be justified by the well-documented immunomodulatory activities of curcuminoids. Curcuminoids have been previously shown to regulate the function of nearly all components of the immune system comprising T and B lymphocytes, monocytes and macrophages, dendritic cells, natural killer cells, neutrophils, eosinophils, and mast cells [35]. Aside from immunomodulatory effects, another mechanism that could account for the observed effects of curcuminoids is mitigation of systemic oxidative stress that usually accompanies adipogenesis and obesity [36, 37]. Curcumin is a well-known antioxidant which is capable of scavenging as well as downregulating the production of reactive oxygen species (ROS) [38]. In a recent study on the same subjects as used in the present trial, we were able to detect a significant reduction in the prooxidant-antioxidant balance index following curcumin therapy, indicating amelioration of systemic oxidative stress burden by this phytochemical [17]. Aside from ROS, curcumin can favorably affect the expression and/or activity of several components of the oxidant-antioxidant system including nitric oxide synthase [39], heme oxygenase 1 [40], glutathione peroxidase (60) and [41], superoxide dismutase [41, 42], catalase [41, 42], free fatty acids [43], paraoxonase [43], and glutathione reductase. Finally, interesting evidence exists as to the modulating effects of curcumin on adipokines, which are important drivers of metabolism and severely dysregulated in obesity [17]. There are several issues that add to the strength of the present study. First, this trial represents the first cross-over study on the anti-inflammatory properties of curcumin. The unique design of this study negates the potential interference of several sources of bias. Second, although the alteration in circulating levels of cytokines has been investigated in a number of clinical studies, there have been only few reports to evaluate the full cytokine profile following curcumin supplementation. Given the complex interplay between the activities and functional properties of pro-inflammatory and anti-inflammatory cytokines, analysis of full cytokine profile would provide a broader view as to the pattern of changes following curcumin therapy. Third, the dropout rate in the current trial was very low which reveals the compliance of participants with their treatment, as well as the robustness of study protocol. Finally, the curcuminoid formulation that was used in the present study contained piperine, a bioactive alkaloid extracted from the *Piper* species which has been shown to serve as an effective absorption enhancer and increase the bioavailability of several therapeutic drugs and nutritional supplements including herbal extracts, water- and fat-soluble vitamins, amino acids, minerals, and antioxidants [44]. Notably, the efficacy of bioperine coadministration in improving the bioavailability of curcumin has been previously reported in a clinical pharmacokinetic study, indicating enhancement by about 2000% [45].

In spite of the aforementioned advantageous, some limitations should also be taken into account prior to the interpretation of results. The most important limitation relates to the rather short duration of curcumin supplementation. Future studies are recommended to look at the cytokine changes, especially those that were found to be significantly affected, over a longer period of time and also compare the magnitude of changes with those of standard anti-inflammatory agents, namely, nonsteroidal anti-inflammatory drugs and corticosteroids. Another limitation of the present trial was lack of controlling for diet during the study. Since interindividual differences in macro- and micronutrient intake may influence the inflammatory status, it would be ideal to address such heterogeneities through implementation of food records and diet diaries.

## 5. Conclusions

In summary, findings arising from the present trial provided additional evidence with respect to the modulatory effects of curcumin on serum levels of IL-1*β*, VEGF, and IL-4. Further and larger randomized controlled trials are warranted to elucidate if the lack of significant alteration in the circulating levels of other evaluated cytokines is due to the ineffectiveness of curcumin or study limitations such as small study population, low dosage, and short duration of supplementation.

## Figures and Tables

**Table 1:** Effect of curcumin on serum cytokine concentrations.

	Study group	*N*	Period				Period effect	Carryover effect	Treatment effect
			1		2				
			Baseline	Endpoint	Baseline	Endpoint			
IL-1*α* (pg/mL)	Curcumin-placebo	15	0.87 ± 1.60	0.62 ± 0.17	0.54 ± 0.12	0.64 ± 0.27	0.23	0.27	0.58
	Placebo-curcumin	15	0.60 ± 0.22	0.59 ± 0.15	0.56 ± 0.10	0.55 ± 0.14			
IL-1*β* (pg/mL)	Curcumin-placebo	15	1.04 ± 0.99	0.60 ± 0.28	0.63 ± 0.24	0.65 ± 0.47	0.31	0.13	0.042
	Placebo-curcumin	15	0.64 ± 0.21	0.66 ± 0.20	0.62 ± 0.21	0.60 ± 0.18			
IL-2 (pg/mL)	Curcumin-placebo	15	4.90 ± 3.56	3.41 ± 2.35	3.31 ± 1.27	3.32 ± 2.02	0.52	0.84	0.22
	Placebo-curcumin	15	3.27 ± 2.12	2.71 ± 0.39	4.06 ± 2.99	3.32 ± 2.25			
IL-4 (pg/mL)	Curcumin-placebo	15	2.81 ± 2.22	1.83 ± 0.39	2.22 ± 1.75	2.01 ± 1.58	0.86	0.01	0.008
	Placebo-curcumin	15	1.50 ± 0.38	2.15 ± 0.63	1.83 ± 0.60	1.74 ± 0.52			
IL-6 (pg/mL)	Curcumin-placebo	15	2.04 ± 1.10	1.68 ± 1.39	1.78 ± 0.90	1.70 ± 0.95	0.32	0.65	0.76
	Placebo-curcumin	15	1.97 ± 3.58	0.99 ± 0.68	1.17 ± 0.35	1.10 ± 0.36			
IL-8 (pg/mL)	Curcumin-placebo	15	10.61 ± 9.43	4.75 ± 2.98	5.24 ± 1.95	5.50 ± 1.60	0.08	0.40	0.69
	Placebo-curcumin	15	9.94 ± 7.02	3.88 ± 2.51	4.44 ± 1.94	4.38 ± 1.55			
IL-10 (pg/mL)	Curcumin-placebo	15	1.44 ± 1.98	0.85 ± 0.43	1.08 ± 1.35	1.00 ± 1.34	0.51	0.10	0.82
	Placebo-curcumin	15	1.69 ± 1.64	1.31 ± 1.56	0.79 ± 0.17	1.00 ± 1.15			
IFN*γ* (pg/mL)	Curcumin-placebo	15	0.97 ± 0.64	0.66 ± 0.65	1.02 ± 1.64	0.86 ± 0.81	0.92	0.47	0.63
	Placebo-curcumin	15	0.39 ± 0.45	0.46 ± 0.27	0.28 ± 0.28	0.41 ± 0.41			
EGF (pg/mL)	Curcumin-placebo	15	191.10 ± 81.75	100.15 ± 55.10	130.19 ± 72.62	182.21 ± 102.10	0.14	0.42	0.73
	Placebo-curcumin	15	190.85 ± 51.12	75.11 ± 54.66	114.68 ± 104.85	118.45 ± 87.13			
VEGF (pg/mL)	Curcumin-placebo	15	166.03 ± 99.04	122.89 ± 97.99	104.88 ± 74.79	151.71 ± 90.13	0.01	0.40	0.03
	Placebo-curcumin	15	177.32 ± 98.72	96.43 ± 43.27	115.25 ± 73.38	122.36 ± 72.39			
MCP-1 (pg/mL)	Curcumin-placebo	15	159.19 ± 78.37	174.02 ± 63.94	171.51 ± 70.00	201.59 ± 59.04	0.09	0.35	0.54
	Placebo-curcumin	15	164.05 ± 70.63	117.51 ± 72.51	131.40 ± 54.03	154.99 ± 80.2703			
TNF*α* (pg/mL)	Curcumin-placebo	15	2.63 ± 2.81	1.50 ± 0.69	1.33 ± 0.64	1.52 ± 0.82	0.64	0.33	0.42
	Placebo-curcumin	15	2.72 ± 1.17	0.92 ± 0.29	1.29 ± 0.92	1.09 ± 0.46			

Values are expressed as mean ± SD. IL: interleukin; EGF: epidermal growth factor; VEGF: vascular endothelial growth factor; MCP-1: macrophage chemoattractant protein 1; TNF*α*: tumor necrosis factor *α*.
